# Impact of functional re-education and environmental adaptation in cancer patients with respiratory pathology: Study protocol

**DOI:** 10.1371/journal.pone.0313207

**Published:** 2025-01-09

**Authors:** Eduardo Jose Fernandez-Rodriguez, Maria Isabel Rihuete-Galve, Alberto Garcia-Martin, Celia Sanchez-Gomez, Fausto Jose Barbero-Iglesias, Roberto Mendez-Sanchez, Ana Silvia Puente-Gonzalez, Susana Saez-Gutierrez, Emilio Fonseca-Sanchez, Juan Jesus Cruz-Hernandez

**Affiliations:** 1 Department of Nursing and Physiotherapy, University of Salamanca, Salamanca, Spain; 2 Institute of Biomedical Research of Salamanca (IBSAL), Salamanca, Spain; 3 Medical Oncology Service, University Health Care Complex of Salamanca, Salamanca, Spain; 4 Department of Labour Law and Social Work, University of Salamanca, Salamanca, Spain; 5 Department of Developmental and Educational Psychology, University of Salamanca, Salamanca, Spain; 6 Department of Medicine, University of Salamanca, Salamanca, Spain; Stanford University School of Medicine, UNITED STATES OF AMERICA

## Abstract

**Background:**

In recent years, cancer survival rates have increased exponentially. However, this rise in survival comes with a significant drawback. As the number of treatment lines has grown, so too have the side effects, which can severely impact patients’ functionality and quality of life. One of the most concerning effects is dyspnoea, a serious health issue that imposes substantial limitations on individuals. While traditional clinical approaches, primarily focused on pharmacological interventions, are commonly employed to manage dyspnoea, we argue that these methods may not always provide optimal symptomatic relief. Therefore, we propose a study protocol to implement an interdisciplinary intervention for these patients. This protocol aims to enhance standard clinical practice by introducing a program of functional re-education and environmental adaptation. We believe this intervention is essential for the follow-up care of patients with respiratory conditions after hospital discharge.

**Methods:**

A two-arm, randomized, parallel, controlled clinical trial will be conducted by the University of Salamanca, Spain, in collaboration with the Medical Oncology Service of the University Health Care Complex of Salamanca. The trial aims to recruit 80 oncology patients who exhibit symptoms of dyspnoea during hospital admission. Participants will be randomly assigned to one of two groups: the control group, which will receive a health education program, and the experimental group, which will receive both the health education program and a functional re-education and environmental adaptation program. Assessments will be conducted at three time points: baseline (prior to hospital discharge), follow-up (15 days after discharge), and the end of the intervention (1 month after discharge). During these assessments, sociodemographic data will be collected, and the following scales will be administered: Barthel Index, Medical Research Council Dyspnoea Scale (mMRC), EuroQol 5-D questionnaire (EQ-5D), Visual Analogue Scale (VAS), Short Physical Performance Battery (SPPB), Tampa Scale for Kinesiophobia (TSK), and the Zarit Reduced Caregiver Burden Scale.

**Discussion:**

The findings of this research can be easily implemented in clinical settings by introducing a targeted intervention to improve the quality of life for this patient population. This study aims to advance traditional clinical approaches by facilitating the recovery or adaptation of cancer patients in their daily routines, in relation to the severity of their symptoms. Our primary goal is to enhance independence and functionality, ultimately improving their overall standard of living.

**Trial registration:**

ClinicalTrials.gov; ID: NCT06035263. Registered on: September 19, 2023. https://register.clinicaltrials.gov/prs/app/action/SelectProtocol?sid=S000DM2U&selectaction=Edit&uid=U0004OJ7&ts=6&cx=f7qqxo.

## Background

In recent years, advancements in oncological treatments, coupled with a strong focus on preventive strategies, have led to earlier diagnoses and a deeper understanding of cancer pathology, resulting in a significant increase in cancer patient survival rates [1]. All of this has led to the increasing importance of the concept of "long survivorship".

Along with this increase in survival and the resulting increase in the number of lines of treatment used, there has been an increase in side effects that negatively impact aspects such as functionality or quality of life in patients [2]. Some of these effects may include tumour asthenia, anxiety or associated respiratory pathology (dyspnoea) [3]. In some patients with advanced cancer, dyspnoea may be a clinical sign of end-stage disease [4]. Approximately 41% of palliative care patients have dyspnoea and 46% of these describe it as moderate or severe [5, 6]. Furthermore, this incidence increases significantly in patients with an anatomopathological diagnosis of lung cancer, reaching incidences of more than 73% in various studies [6].

Most patients view dyspnoea as an uncontrollable limitation, which leads to avoidance behaviours that further increase their inactivity. This inevitably negatively impacts their functionality [7] and results in patterns of fear and avoidance of exercise similar to those observed in patients with chronic pain, chronic fatigue syndrome, or fibromyalgia [8, 9]. Patients with respiratory problems adapt to their symptoms by reducing their activity levels. This leads to a deterioration in physical fitness and exertional dyspnoea, known as the "respiratory patient cycle" [10–13].

For managing dyspnoea, we acknowledge that conventional clinical practices, primarily based on pharmacological treatments, can be applied. However, we believe these measures alone are insufficient for achieving optimal symptom control.

Current pharmacological treatments often fall short in managing the primary symptom of respiratory pathologies: dyspnoea, likely due to its multifactorial nature. Most patients perceive dyspnoea as an uncontrollable limitation, leading them to adopt avoidance behaviours that further reduce their activity levels and negatively impact their functionality. Therefore, from a comprehensive care perspective, it is crucial to employ additional interventions that help patients readjust to their daily activities, rather than focusing solely on symptom management.

We have observed that cancer patients with respiratory issues often reduce their activity levels in response to their symptoms. This reduction leads to a decline in physical condition and increased dyspnoea with exertion. Over time, dyspnoea contributes to progressive disability, resulting in decreased mobility, reduced self-esteem, and impaired work and social relationships.

These data show that associated respiratory pathology is a common problem in oncology, which is still underestimated by professionals [14–17]. It has been observed that people with this process find it difficult to normalise their daily lives, either because of a deterioration in their clinical condition or because of a problem in generalising what they have learned during their hospital stay.

In terms of intervention, the NCCN panel considers education and energy conservation techniques to be fundamental, always within a complete functional rehabilitation programme [18, 19].

In addition to educational measures, the NCCN panel recommends the prescription of energy conservation techniques, presenting them as useful in controlling this associated respiratory pathology. To this end, the panel analyses, on the one hand, a multicentre clinical trial involving 296 patients currently under active treatment, in whom a reduction in symptom intensity was reported after application of the programme [20]. On the other hand, they present a meta-analysis including 113 studies with a total sample of 11,525 patients, showing an improvement in individuals after the use of non-pharmacological measures to control associated respiratory pathology (weighted effect size, 0.30; 95% CI, 0.25–0.36; p<0.001).

The common presence of associated respiratory pathology in cancer patients is a factor that can cause changes in body composition, such as loss of muscle mass or an increase in visceral fat [21, 22]. This type of non-pharmacological intervention has been shown to be an important tool in improving symptoms and some parameters related to body composition [23]. In the study by Madison et al, moderate exercise (bioelectrical impedance) over 12 weeks was associated with a reduction in body fat in colorectal cancer survivors [24]. Another study showed that an 8-week exercise intervention increased skeletal muscle mass and decreased visceral fat in a group of head and neck cancer patients undergoing chemotherapy [25]. Fernandez-Lao et al showed that a multimodal exercise programme in breast cancer patients led to a reduction in body fat and an increase in lean body mass in a group of breast cancer patients [26]. However, despite the potential effects, there is little evidence of functional rehabilitation interventions that have assessed the effects on body composition in patients with associated respiratory pathology.

Scientific evidence and recent expert consensus on cancer care highlight the benefits of psychosocial interventions in both preventing and treating various aspects of cancer. These interventions have demonstrated efficacy in improving physical function, reducing fatigue, enhancing quality of life, and addressing pain, anxiety, and depressive symptoms among cancer survivors and patients undergoing treatment [27].

Non-pharmacological psychosocial interventions have therefore been shown to be even more effective than pharmacological interventions in addressing these associated symptoms, leading us to consider the bio-psychosocial approach and multidisciplinary intervention (oncology, nursing, physiotherapy, occupational therapy and medicine) as the global context of intervention [27, 28]. For this reason, aspects related to loss of function and pain associated with anxiety-avoidance disorders should also be assessed and addressed, which can be assessed using kinesiophobia scores [29].

The cognitive-behavioural model of fear of exercise suggests that patients with chronic pain or fatigue syndrome tend to avoid activity because they believe that activity is the cause of these symptoms, such as pain and fatigue. Avoidance behaviour leads to even greater fear and symptoms, resulting in more pain or fatigue [29, 30], which may extend to patients with associated respiratory pathology, so it is important to restore optimal activity levels and avoid loss of physical function and ability [29].

The selection of the most suitable intervention setting depends on the clinical complexity and the patient’s capacity to self-manage their condition. Consequently, and with expert support, our study proposes a supervised home-based intervention following hospital discharge. This approach is tailored to the specific needs of patients with respiratory pathology and aligns with recommendations aimed at enhancing access to and adherence to functional rehabilitation programs [27–31].

Supervised follow-up outside the healthcare setting, both in the community and at home, has been shown to be successful in previous trials. This is shown in a meta-analysis of 14 randomised controlled clinical trials in breast cancer survivors with supervised intervention by telephone or e-mail [32]. In any case, despite the choice of the home setting for its feasibility in our study population, supervision and controlled follow-up of the intervention is still a guarantee of good results, as shown in another recent meta-analysis of 128 trials with a total of 13,050 cancer patients, where supervised programmes had greater effects on physical activity [33]. Follow-up is important not only for the correct implementation of the intervention programme, but also for achieving high adherence to the programme, as shown in a review of 23 trials and 1372 patients [34].

The best results in patients surviving or undergoing cancer treatment have been obtained with multimodal exercise programmes that combine different types of exercise, mainly aerobic and strength training [27, 28, 35], in addition to other interventions such as re-education in activities of daily living and health education, adapted to the patient’s general condition and functional capacity [10, 21, 27].

For this reason, we propose an interdisciplinary intervention involving occupational therapists, nurses, and doctors specializing in this patient population. This approach aims to enhance conventional clinical practice by incorporating a program of functional re-education and environmental adaptation. We believe this intervention is essential for the follow-up care of patients with respiratory pathology after hospital discharge.

As noted, associated respiratory pathology is one of the most prevalent and persistent symptoms related to cancer and its treatment, significantly impacting quality of life [12, 13]. The results of this study could be swiftly integrated into patient care processes to help normalize their daily lives. This research project aligns with the Regional Research and Innovation Strategy for Smart Specialization (RIS3) of Castilla y León, which prioritizes health and social care, demographic change, and improved quality of life. It aims to develop new strategies for facilitating independent living at home for chronic and dependent patients.

The proposed intervention involves professionals from various levels of care, creating a multidisciplinary team to support its development and implementation. By implementing this intervention outside the hospital setting, we aim to alleviate the burden on local clinical practices. This approach benefits patients by continuing their rehabilitation while reducing their exposure to infections and minimizing the strain on their environment. Additionally, it benefits the hospital system by effectively addressing patient needs outside of the care center, which is currently overwhelmed due to multiple factors.

### Aim

#### HYPOTHESIS

Functional re-education and environmental adaptation programs enhance fatigue, pain, functional capacity, and quality of life in oncology patients with associated respiratory pathology.

#### OBJECTIVES

The primary objective of the study is to evaluate the impact of a functional re-education and environmental adaptation program on the autonomy of oncology patients with associated respiratory pathology.

The secondary objectives are as follows:

1. To assess the effect of a functional re-education and environmental adaptation program on activities of daily living, attention, executive functions, and quality of life parameters in oncology patients with associated respiratory pathology.
2. To evaluate the impact of a functional re-education and environmental adaptation program on physical parameters in oncology patients with associated respiratory pathology, including fatigue, pain, functional capacity, and body composition.
3. To examine the correlation between the level of associated respiratory pathology and the level of dependency in individuals.
4. To explore the relationship between the level of dependency and the level of kinesiophobia in individuals.
5. To determine if there are differences in effectiveness based on the oncological anatomopathological diagnosis.

## Material and methods

### Design and setting of the study

Randomised, stratified, prospective, longitudinal clinical trial with parallel fixed-assignment experimental and control groups.

### Sample/participants

#### Participants

The reference population comprises cancer patients with associated respiratory pathology who are admitted at the time of enrollment. Participants will be selected from this population through consecutive sampling based on the following criteria.

#### Study setting

The study will be conducted in a combined setting involving the specialized care unit of the University Health Care Complex of Salamanca, CAUSA (Medical Oncology Service), and the University of Salamanca (Occupational Therapy Teaching and Care Unit, UDATO).

SELECTION CRITERIA FOR PARTICIPANTS:

Inclusion criteria:

- To have, among the reasons for admission, an anatomopathological diagnosis of newly diagnosed or relapsed oncological disease.
- To be admitted to the Oncology Department of the University Hospital of Salamanca.
- Moderate to severe dependency: Barthel Index score between 20 and 55 points.
- Sign an informed consent form authorising their voluntary participation in the study.

Exclusion criteria:

- Cognitive impairment as assessed by the Mini-Mental State Examination (MMSE) of less than 24 points.

Withdrawal criteria:

- Patient death.
- Disease progression to a terminal state.
- Hospitalisation of the patient at the time of home follow-up.
- No final assessment.

#### Randomization

Among the patients in the study population, those who meet the selection criteria following an initial interview with the research team will be randomized into two groups: an intervention group (IG) and a control group (CG). The allocation sequence will be generated by an independent researcher using Epidat 4.2 software, with a 1:1 ratio. Randomization will be based on the order in which participants complete the baseline assessment, and the randomization sequence will remain concealed until each patient is assigned to their respective group.

#### Blinding

The tasks of sequencing, randomization, recruitment, and assignment of senior centers to each group (experimental or control) will be handled by research staff who are not involved in the assessments or interventions for either group, thereby minimizing potential bias. Participants will also be blinded and will not know which group they have been assigned to or which intervention they will receive.

To reduce the risk of contamination between groups, the assessment process will be conducted by external research staff who have been trained to avoid subjective bias. These assessors will be unaware of the intervention group to which each senior center is assigned, ensuring that the clinical trial maintains masking of the blinded assessment by third parties. Additionally, the researchers responsible for the statistical analysis of the trial will be blinded to further enhance the rigor and scientific quality of the study.

#### Sample size

The sample size was estimated based on the potential change in one of the main variables of the study, the Barthel Scale score. For this purpose, the results of a pilot study with similar characteristics using a sample of the oncology population were used as a reference. In this study, the Barthel Scale score was modified by 5.8 points. With these assumptions, assuming an alpha risk of 0.05 and a beta risk of 0.2 in a bilateral contrast, 40 subjects in the first group and 40 subjects in the second group are required to detect a difference equal to or greater than 5.8 units. The common standard deviation is assumed to be 8.7. A loss to follow-up rate of 10% was estimated. Sample size estimation was performed using EPIDAT 4.2 software.

### Procedures and data collection

#### Evaluations and study plan

**Fig 1:**
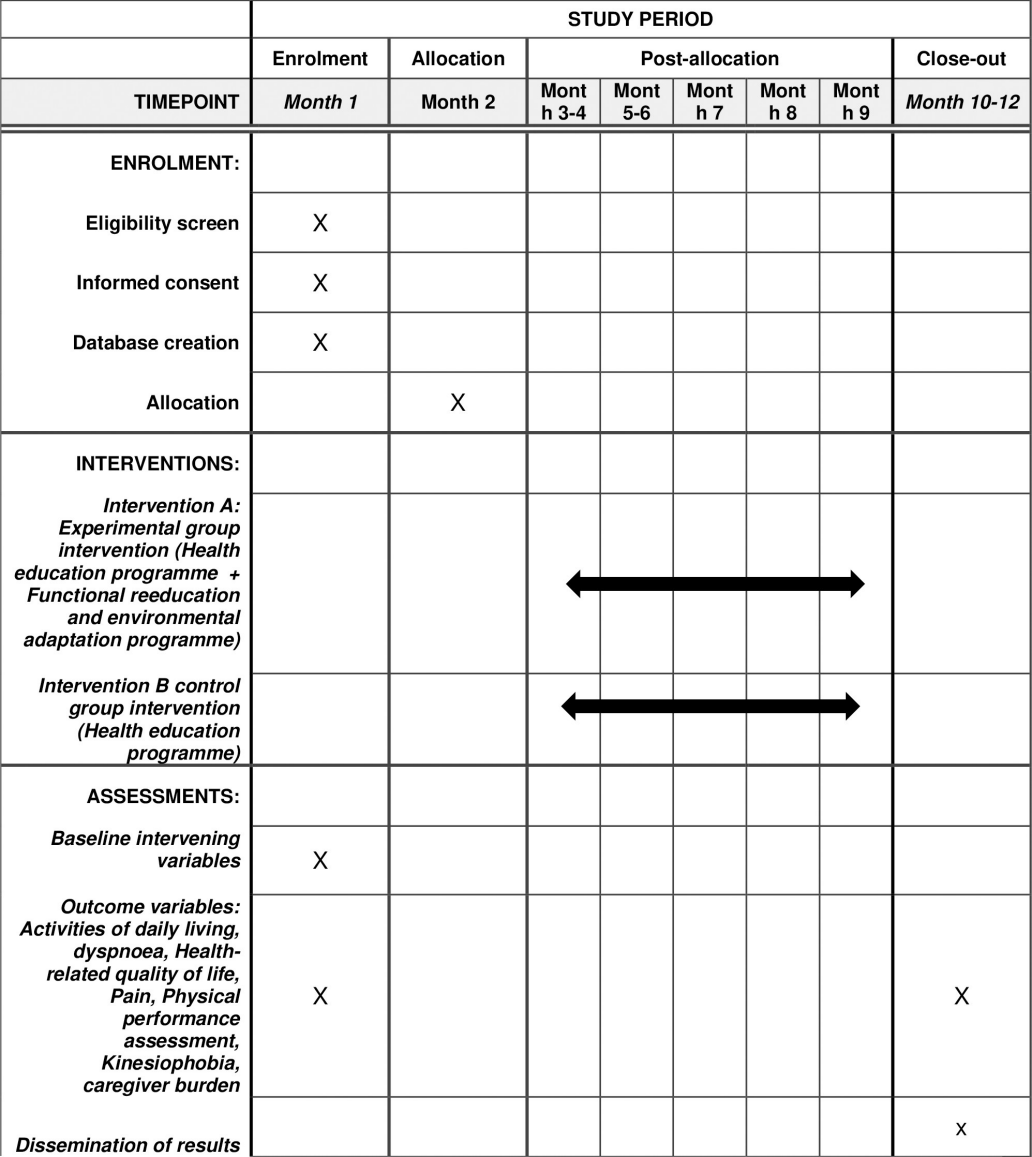
The schedule of enrolment, interventions, and assessments of SPIRIT 2013.

Participants will undergo three assessments during the study: the baseline assessment at the start (upon patient referral and before hospital discharge), a follow-up assessment (15 days later), and a final assessment (one month after the baseline assessment, following the completion of the intervention, as shown in Fig 1).

**Fig 2:**
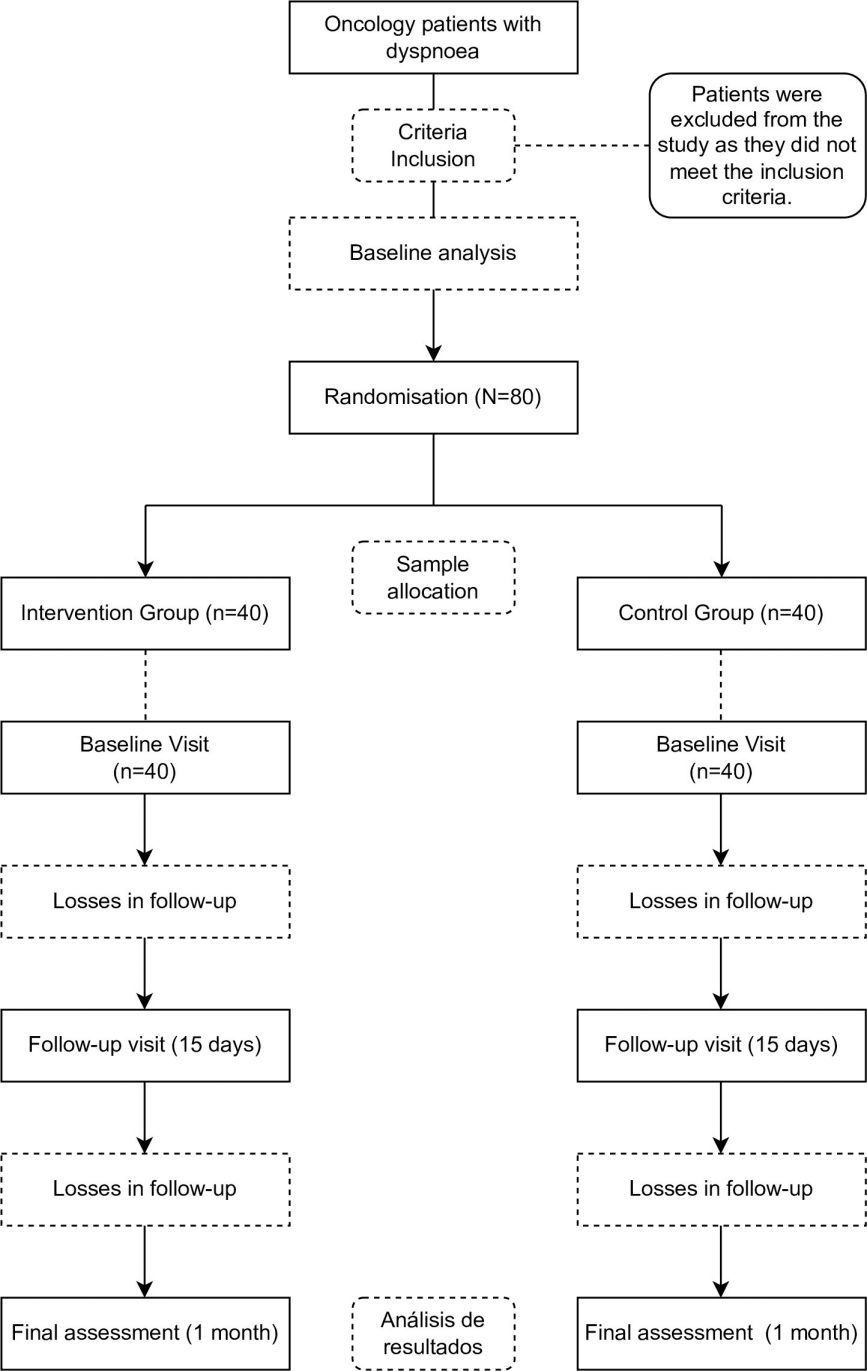
Study methods flow chart.

The baseline assessment will be conducted after recruitment and before participants are randomized into their respective groups. This initial assessment will record the independent and primary outcome variables under study, as well as any intervening variables, using various objective tests. Following randomization, the intervention will be administered to each group. Subsequently, two additional evaluations will be performed: a follow-up assessment and a final assessment, where participants will complete the same objective tests as in the initial evaluation. Study findings will be communicated through a personalized report to individuals who request this information. All assessments will be carried out by the research team, who have been trained and instructed in the evaluation process. A flow chart outlining the study methodology is presented in Fig 2 for further details.

#### Description of the variables/Tools employed in the evaluation of the variables

*Main Outcome Variable*. *Activities of Daily Living (ADLs)*: The primary outcome will be the level of dependency in performing ADLs, assessed using the Barthel Index (BI). The BI is a widely recognized tool for measuring a person’s ability to perform basic activities necessary for daily living, such as feeding, bathing, grooming, dressing, bowel and bladder control, toilet use, transfers (e.g., from bed to chair), mobility, and stair use. The Spanish version of the BI, which has been translated and validated, will be utilized for this study. Each of the ten items on the BI scale is scored based on the degree of independence, with scores ranging from 0 to 15 points per item. The total possible score ranges from 0 (completely dependent) to 100 (completely independent), providing a quantifiable measure of the patient’s functional status [36–38].

*Secondary Outcome Variables*. *Assessment of Associated Respiratory Pathology or Dyspnoea*: Dyspnoea, a common symptom in patients with respiratory pathology, will be evaluated using the Medical Research Council Dyspnoea Scale (mMRC). The mMRC scale allows patients to self-assess the severity of their dyspnoea in a straightforward and visual manner. It classifies dyspnoea into five levels of severity, ranging from Grade 0 (no dyspnoea except with strenuous exercise) to Grade 4 (too dyspnoeic to leave the house or breathlessness when dressing/undressing). This scale is a reliable tool for quantifying the impact of dyspnoea on a patient’s daily activities [39].

*Health-Related Quality of Life (HRQoL) Assessment*: HRQoL will be assessed using the EuroQol 5-Dimension (EQ-5D) questionnaire. The EQ-5D is a standardized instrument that evaluates five dimensions of health: mobility, self-care, usual activities, pain/discomfort, and anxiety/depression. Each dimension is rated on three levels: no problems, some problems, and extreme problems. An adapted version validated for the Spanish population will be employed. Additionally, the EQ-5D includes a Visual Analogue Scale (VAS), where patients rate their overall health on a scale from 0 (worst imaginable health) to 100 (best imaginable health). This comprehensive measure provides insight into the patient’s perceived health status [39].

*Pain Assessment*: Pain intensity will be measured using the Visual Analogue Scale (VAS). The VAS is a simple and widely used tool for assessing pain, where patients indicate their pain level on a 10 cm horizontal line. The endpoints are labeled as "no pain" (0) and "worst pain imaginable" [10]. This scale is particularly effective for capturing changes in pain intensity over time [39].

*Physical Performance Assessment*: Physical performance will be evaluated using the Short Physical Performance Battery (SPPB). The SPPB is designed to assess lower extremity function through a series of tests, including balance, gait speed, and chair stands. This battery is validated in primary care settings and has been shown to predict outcomes such as disability, dependency, institutionalization, and mortality. Scores range from 0 to 12, with higher scores indicating better physical performance [40].

*Assessment of Pain/Fatigue-Related Fear of Movement*: The Tampa Scale for Kinesiophobia (TSK) will be used to assess the fear of movement related to pain or fatigue. The modified 11-item version of the TSK (TSK-F), validated for use in oncology patients, will be employed. This scale measures the degree of fear associated with movement, which can be a significant barrier to physical rehabilitation. Each item is rated on a 4-point Likert scale, with higher scores indicating greater fear of movement [41, 42].

*Assessment of Caregiver Burden*: Caregiver burden will be assessed using the Zarit Reduced Caregiver Overload Scale. This is a shortened version of the original Zarit Burden Interview, developed by Gort A et al. in 2005. The scale consists of 7 items, each scored on a Likert scale from 0 (never) to 5 (nearly always). Scores above 17 indicate a significant caregiver burden, often referred to as "familial claudication." This measure is essential for understanding the impact of the patient’s condition on their primary caregiver.

Other variables to be studied: care support at home, religious and spiritual practices, BMI, range of movement, the muscle strength.

*Data Collection and Management*. An individual data collection sheet will be created for each participant, capturing all relevant variables. Data will be systematically recorded and stored in a purpose-built database designed for this study. This approach ensures the accurate tracking of participant progress and the reliable analysis of study outcomes.

#### Interventions

To describe the intervention, we need to distinguish between experimental and control groups. We also need to describe that the research team will consist of occupational therapists, nurses, physiotherapists and medical oncologists. Each professional will be responsible for their part of the work:

Interventions will be presented in accordance with the TIDieR standards.

CONTROL Group (CG): Health education programme.

Short name: Health education programme.

Why: At the time of hospital discharge, at the end of the baseline assessment, the control group will receive instructions and recommendations for maintaining an active and healthy lifestyle as part of a health education program. This program will emphasize the benefits of an active lifestyle, provide general guidelines, and highlight the importance of nutrition and hydration in maintaining a healthy life.

What (materials): The only material required for reflex locomotion is an informational pamphlet.

What (procedures): Instructions and recommendations for maintaining an active and healthy lifestyle will be provided as part of the health education program. Participants will receive a dossier containing these instructions and recommendations, similar to the experimental group.

Who will carry out the interventions: All interventions will be conducted by a qualified occupational therapist with over 10 years of experience in treating oncology patients.

How: The material will be given to each patient individually at the time of referral, prior to their hospital discharge.

Where: All sessions will be held at the Medical Oncology Service of the University Health Care Complex of Salamanca and the Occupational Therapy Teaching Unit of the University of Salamanca.

When and for how long: The intervention will consist of a single session. Details regarding the time and duration of the session will be provided to participants.

Adaptation: Adaptation measures are available to address the diverse topics and individual needs of the intervention. Sessions will be tailored to each topic, though current assessment of the therapy’s effectiveness is not yet available.

How well (planned): Supervision of the therapy will occur through weekly meetings between therapists and researchers.

INTERVENTION Group (IG): Functional re-education and environmental adaptation programme.

Short name: Functional re-education.

Why: The program is based on the principle of adapting the environment to meet the needs of individuals with disabilities. It considers two key factors: the individual’s ability to perform daily tasks and the environmental adjustments needed to facilitate a more normalized lifestyle. Pharmacological therapies offer only partial symptomatic relief for dyspnoea, leading patients to limit their activities to avoid worsening symptoms. Our program aims to maximize functional gains, reduce the risk of future complications, and help individuals maintain their independence in daily activities.

What (materials): To support autonomy, a range of materials and equipment will be necessary for the intervention. These may include walkers, pedicabs, canes, wheelchairs, and other assistive devices for daily activities. The selection of materials and equipment will be tailored to each patient’s specific needs.

What (procedures): Patients in the intervention group will participate in a supervised and structured program at home. This program will combine health education with functional re-education and environmental adaptation. It will include guidance on activities of daily living and the use of assistive devices and environmental modifications. The intervention will last for one month, starting from the baseline assessment at the time of hospital discharge.

1. RE-EDUCATION IN ACTIVITIES OF DAILY LIVING (AVD)

Specific training will be conducted after the assessment and before patients are discharged from the University Health Care Complex. This training aims to identify factors that interfere with the performance of daily activities. The intervention will consist of three components: Direct intervention to improve Activities of Daily Living (ADLs), instruction on Energy Saving Techniques (ESTs), which focus on simplifying activities and guidance on hygiene measures to address sleep disorders, in line with the National Comprehensive Cancer Network’s specific guidelines [43].

The intervention consists of three parts:

- Direct intervention on Activities of Daily Living (ADLs), delivered in the hospital and transferable to the home environment. The aim is to teach patients to be as autonomous as possible. The first session takes place the day before the patient is discharged from hospital, and supervision continues for the remaining month of the intervention.
- Training in energy saving techniques (EST), based on the simplification of activities.
  1. Organise the work areas in which the activity is carried out.
  2. Work plans must be adapted and care must be taken to ensure that objects are within the patient’s reach.
  3. Where possible, the activity should be carried out in a sitting position and as soon as possible in an upright position.
  4. Impulsive and forceful movements should be transformed into studied, slow and harmonious movements.
  5. Heavy and light activities should be alternated, with periods of rest in between.
  6. Periods of balanced activity should be combined with periods of rest.
  7. Avoid movements or activities that cause exertional dyspnoea.
- Advise on hygiene measures for sleep disorders as outlined in the National Comprehensive Cancer Network specific guidelines.
  - Maintain as regular a sleep-wake cycle as possible, especially at waking.
  - Avoid stimulants and other substances before bedtime.
  - Maintain good nocturnal analgesic control, preferably with long half-life analgesics.
  - Avoid unnecessary time in bed during the day; for bedridden patients, provide physical and cognitive stimulation throughout the day.
  - Minimise night-time disturbance from noise, medication administration or other environmental conditions.
  - Remove unpleasant stimuli, such as clocks, from the bedroom.
  - Avoid lying awake in bed at night trying to sleep; do some relaxing activity (reading), preferably outside the bed, until sleepiness sets in.
  - Avoid late afternoon naps.
  - Use hypnotic medication after a proper assessment of the sleep disorder and avoid overuse.

2. PRESCRIPTION OF ASSISTIVE DEVICES AND ENVIRONMENTAL ADAPTATIONS

Before the patient is discharged and following the baseline assessment, an evaluation will be conducted to determine the need for assistive devices that could enhance the patient’s autonomy. This assessment will also identify potential barriers in the patient’s home that might impact their independence after discharge. This evaluation will take place on the day before discharge as well as "in situ" at the patient’s home.

Considerations include:

Mobility aids such as canes, walkers, and wheelchairs.

Personal care and protection devices, including anti-decubitus cushions, toilet seat adapters, and other bathroom equipment.

These aids will be prescribed based on the individual needs of the patient to maximize independence.

In addition, the intervention in spirituality will be incorporated as an integral part of our multidisciplinary approach, aiming to enhance the overall well-being and quality of life of patients. Spirituality, understood as a personal resource that can provide meaning, hope, and inner peace, has been shown to positively impact the acceptance of illness and alleviate emotional and physical symptoms. The spiritual intervention will include support sessions that encourage personal reflection and connection with sources of inner strength, tailored to the beliefs and individual needs of each patient. We believe that this intervention can elevate patients’ morale, promote a positive attitude toward treatment, and significantly contribute to their overall well-being.

Who will conduct it: All sessions will be held jointly at the Medical Oncology Service of the University Health Care Complex of Salamanca and the Occupational Therapy Teaching and Care Unit at the University of Salamanca.

How: The intervention will be conducted individually, with each session lasting 45 minutes over a 12-month period.

Where: Sessions will be held at both the Medical Oncology Service of the University Health Care Complex of Salamanca and the Occupational Therapy Teaching and Care Unit at the University of Salamanca.

When and for how long: Participants will receive daily sessions throughout their hospital stay from the time of referral. Each session will last approximately 45 minutes, and participants will follow a consistent weekly schedule. After discharge, personalized home interventions will be provided, along with follow-up visits for evaluations at 15 days and 1 month.

Adaptation: Given the individualized nature of the intervention and the diverse needs of the subjects, sessions will be tailored to each person.

How well (planned): Therapy will be supervised through weekly meetings between therapists and researchers, with close monitoring of participants’ attendance at sessions.

WORK PLAN AND STRUCTURE OF THE VISITS

Once potential candidates are identified at the Oncology Unit of the University Health Care Complex of Salamanca (CAUSA), the researchers will schedule an interview to explain the study’s purpose and obtain their informed consent. Each participant will undergo three assessment visits: a baseline visits before randomization and two follow-up visits, 15 days and 1 month after the baseline visit. Each visit will follow the same structure and last approximately one hour, during which the study variables will be assessed.

Baseline Visit: This visit will occur prior to hospital discharge and will gather baseline data, including sociodemographic information, medical history, presence of comorbidities, and use of concomitant medications. It will also confirm compliance with the selection criteria. All study variables and questionnaires will be assessed and completed. At the end of this visit, participants will be randomly assigned to one of the two study groups. The structure and organization of the functional rehabilitation program will be explained to participants in the experimental group during a dedicated session.

Follow-up and Final Visits: The follow-up visits, scheduled 15 days and 1 month after the baseline visit, will replicate the baseline assessment, excluding sociodemographic data, which will only be collected during the baseline visit. These follow-up visits will take place at the Occupational Therapy Teaching and Care Unit (UDATO) of the University of Salamanca.

In addition, to facilitate the sharing of experiences among patients, we will implement a satisfaction questionnaire specifically designed to gather feedback from participants in the project. This questionnaire will provide valuable insights into their experiences and perceptions, enabling us to support and educate new patients who join the study. While we recognize the importance of peer support, we must acknowledge that organizing group sessions may pose challenges due to the health status of the patients involved, which could limit their ability to participate. Therefore, we believe that utilizing this questionnaire offers an effective alternative to encourage the exchange of experiences while prioritizing the well-being of our patients.

### Ethical aspects

The study will be carried out with the approval of the Ethics Committee for Clinical Research of the Health Region of Salamanca, with the prior informed consent of the subjects and in accordance with the Declaration of Helsinki. Participants will be informed of the objectives of the project and the risks and benefits of the research to be carried out (informed consent). Likewise, the confidentiality of the subjects enrolled will be guaranteed at all times, in accordance with the provisions of Organic Law 3/2018, of 5 December, on the Protection of Personal Data and the Guarantee of Digital Rights, and Regulation (EU) 2016/679 of the European Parliament and of the Council of 27 April 2016 on Data Protection (RGPD), and under the conditions established by Law 14/2007 on Biomedical Research.

Significant changes to the protocol (such as change to the assessment tools, changes to the selection criteria or to the interventions) will be communicated immediately to the Ethics Committee.

As this is a randomised clinical trial, it follows the CONSORT guidelines and has been registered. TRIAL REGISTRATION: ClinicalTrials.gov; ID: NCT06035263

### Data analysis

For the descriptive analysis of the data, normality was tested using the Kolmogorov-Smirnov and Shapiro-Wilk tests (n<30). Normally distributed variables will be defined by mean, standard deviation and interval, while variables that do not follow a normal distribution will be defined by median and interquartile range. The qualitative variables of the study will be defined by frequencies and percentages.

With regard to the quantitative analysis, a correlation analysis (Pearson’s correlation coefficient) will be used to demonstrate the validity of the assessment procedure chosen in our study. Cronbach’s alpha coefficient will be used to demonstrate its reliability. For the comparison of two means, the Student’s t-statistic (parametric test for independent samples), the Mann-Whitney U-statistic (non-parametric tests with two independent samples) and the Wilcoxon t-test (non-parametric tests for repeated means) will be used. The comparison of three or more means has also been used and will be analyzed using the ANOVA test in the situation of independent groups (parametric via Snedecor’s F ANOVA and non-parametric via Kruskal-Wallis’ H). For repeated measures, Snedecor’s F test is used parametrically and Friedman’s test is used non-parametrically. Correlations are carried out using two methods: Pearson’s correlation (normal distribution) or Spearman’s correlation (non-normal distribution). Multivariate logistic regression analysis is used to identify variables associated with events of interest. In the logistic regression model, variables that were significant in the bivariate analysis or that are relevant to the study are included in the logistic regression model. For qualitative or categorical variables, contingency tables and the chi-square test of significance are used when there are two independent samples.

P-values of less than 0.05 will be considered significant, i.e. with a confidence interval of 95%. The statistical software IBM SPSS Statistics version 28.0.1 will be used.

### Rigour

This study protocol has been developed according to the evidence-based recommendations of the 2013 SPIRIT Statement [28] on the minimum content of a clinical trial protocol. The study design also follows the recommendations of the 2010 CONSORT Statement [29] on standards for conducting parallel group randomised controlled clinical trials (RCTs).

## Discussion

The findings of this research can be readily applied in clinical settings by implementing a targeted intervention designed to improve the quality of life for cancer patients. This study aims to advance traditional clinical approaches by facilitating the recovery or adjustment of cancer patients in their daily routines based on the intensity of their symptoms. Our primary goal is to enhance independence and functionality, thereby improving their overall standard of living.

It is crucial to emphasize the importance of promoting such interventions among cancer patients. While interventions aimed at improving survival rates are often prioritized, we argue that these should be complemented by a strong focus on achieving a satisfactory quality of life. Our goal is to address the gaps identified in current clinical practice, where interventions have predominantly focused on symptomatic relief without considering their impact on daily activities. Tailored programs that emphasize targeted training in instrumental activities of daily living are essential.

Furthermore, we believe this intervention will not only aid in the recovery of patient autonomy but also positively impact their environment. The progression of the disease and associated symptomatic deterioration often lead to the environment adapting to take on caregiving roles. Improving patient autonomy can help reduce the caregiving burden on relatives, thereby enhancing their quality of life as well.

It is also important to note the limited research history on the control and symptomatic management of cancer patients, which underscores the need for evidence-based interventions.

### Limitations

The investigation adheres to all CONSORT recommendations. However, due to the intervention’s nature, subjects will not be able to be blinded to it. To limit contamination between groups, the individual carrying out the study measurements at each visit and the statistical analysis will be blinded.

### Dissemination plan

The study’s outcomes will be disseminated with the aim of attaining the utmost visibility. They will be published in scientific journals with open access. The primary findings will be published first, with secondary results expected to follow suit. Additionally, the results will be presented at national and international conferences and seminars.

Three publications resulting from this research have been planned.

- The initial step would involve publishing the study protocol once approval has been obtained from the corresponding CEIC, and registering it on Clinicaltrials.gov.
- The second stage would focus on publishing the primary outcomes of the study, which encompass the effects of the intervention on activities of daily living, fatigue, pain, and quality of life.
- The final phase would concentrate on the secondary outcomes of the intervention on cognitive performance variables.

### How potential changes in the study will be approached

Significant modifications to the protocol (such as change to the assessment tools, modifications to the selection criteria or to the interventions) shall be reported immediately to the bioethics committee for approval.

## Conclusions

This manuscript outlines a study protocol designed to assess the impact of a functional re-education and environmental adaptation program on enhancing the autonomy of oncology patients with associated respiratory pathology.

## Supporting information

S1 File SPIRIT 2013 checklist: Recommended items to address in a clinical trial protocol and related documents. (DOCX)

S2 File Clinical trials registration document. Recruitment for the study has not commenced yet.
(PDF)

S3 File Study protocol for ethics committee. (PDF)
