# Supplementary material for: Impact of functional re-education and environmental adaptation in cancer patients with respiratory pathology: Study protocol
Source: PLoS One. 2025 Jan 9;20(1):e0313207. doi: 10.1371/journal.pone.0313207 (PMC11717268; doi:10.1371/journal.pone.0313207)
Supplement: S2 File — Recruitment for the study has not commenced yet. (PDF) [file pone.0313207.s002.pdf]

ClinicalTrials.gov Protocol Registration and Results System (PRS) Receipt
Release Date: November 1, 2023

ClinicalTrials.gov ID: NCT06035263

Study Identification

Unique Protocol ID: University of Salamanca 2

Brief Title: Effects of a Functional Re-education and Environmental Adaptation Programme in Cancer Patients With Associated Respiratory Pathology

Official Title: Effects of a Functional Re-education and Environmental Adaptation Programme on Levels of Dependency, Dyspnoea, Functional Capacity, Quality of Life and Caregiver Burden in Cancer Patients With Associated Respiratory Pathology

Secondary IDs:

Study Status

Record Verification: November 2023

Overall Status: Not yet recruiting

Study Start: January 1, 2024 [Anticipated]

Primary Completion: December 31, 2024 [Anticipated]

Study Completion: December 31, 2024 [Anticipated]

Sponsor/Collaborators

Sponsor: University of Salamanca

Responsible Party: Principal Investigator
Investigator: Prof. Dr. Eduardo J Fernández Rodríguez [efernandezrodriguez]
Official Title: Professor
Affiliation: University of Salamanca

Collaborators:

Oversight

U.S. FDA-regulated Drug: No

U.S. FDA-regulated Device: No

U.S. FDA IND/IDE: No

Human Subjects Review: Board Status: Approved
Approval Number: 2022/10
Board Name: COMITÉ de Ética de la Investigación
Board Affiliation: University of Salamanca
Phone: 923291100
Email: comite.etico.husa@saludcastillayleon.es
Address:

Paseo de San Vicente, 58-182
37007 Salamanca
Comité Ético de Investigación con Medicamentos

Data Monitoring: No

FDA Regulated Intervention: No

Study Description

Brief Summary: the investigators propose an interdisciplinary intervention, carried out by occupational therapists, nurses, physiotherapists and doctors specialised in this type of patient, aimed at improving conventional clinical practice and implementing a programme of functional re-education and environmental adaptation that implements conventional clinical practice, and which the investigators also consider to be an essential intervention in the follow-up of patients with associated respiratory pathology once they are discharged from hospital.

Detailed Description: In recent years, improvements in oncological treatments, together with a strong commitment to preventive strategies, have led to better early diagnosis and better knowledge of the oncological pathology itself, resulting in an exponential increase in the survival of cancer patients. All of this has led to the increasing importance of the concept of "long survivorship".

Along with this increase in survival and the resulting increase in the number of lines of treatment used, there has been an increase in side effects that negatively impact aspects such as functionality or quality of life in patients. Some of these effects may include tumour asthenia, anxiety or associated respiratory pathology (dyspnoea). In some patients with advanced cancer, dyspnoea may be a clinical sign of end-stage disease. Approximately 41% of palliative care patients have dyspnoea and 46% of these describe it as moderate or severe. Furthermore, this incidence increases significantly in patients with an anatomopathological diagnosis of lung cancer, reaching incidences of more than 73% in various studies.

Most patients perceive this dyspnoea as a limiting factor beyond their control, leading to avoidance behaviours that further increase their inactivity. This inevitably has a negative impact on their functionality and leads to patterns of fear/avoidance of exercise in cancer patients, as seen in patients with chronic pain, chronic fatigue syndrome or fibromyalgia. Patients with respiratory problems adapt to their symptoms by reducing their activity levels. This leads to a deterioration in physical fitness and exertional dyspnoea, known as the "respiratory patient cycle".

For the control of dyspnoea, the investigators believe that the measures used in conventional clinical practice, developed mainly from a pharmacological point of view, can be implemented, but that they are not sufficient to achieve optimal symptomatic control.

Current pharmacological treatment does not always manage to control the main symptom of respiratory pathologies: dyspnoea, perhaps because of its multifactorial nature. Most patients perceive this symptom as a limiting factor beyond their control, leading them to adopt avoidance behaviours that increase their inactivity, with negative consequences for their functionality. Therefore, from the point of view of comprehensive care for the patient, not only for the symptom, the investigators have observed that it is necessary to use other interventions that achieve the patient's readaptation to their daily activity.

the investigators have observed that cancer patients with respiratory pathology adapt to their symptoms by reducing their level of activity. This leads to a deterioration in their physical condition and dyspnoea on exertion. Dyspnoea leads to progressive disability with loss of mobility, self-esteem, work and social relationships.

These data show that associated respiratory pathology is a common problem in oncology, which is still underestimated by professionals. It has been observed that people with this process find it difficult to normalise their daily lives, either because of a deterioration in their clinical condition or because of a problem in generalising what they have learned during their hospital stay.

In terms of intervention, the NCCN panel considers education and energy conservation techniques to be fundamental, always within a complete functional rehabilitation programme.

In addition to educational measures, the NCCN panel recommends the prescription of energy conservation techniques, presenting them as useful in controlling this associated respiratory pathology. To this end, the panel analyses, on the one hand, a multicentre clinical trial involving 296 patients currently under active treatment, in whom a reduction in symptom intensity was reported after application of the programme. On the other hand, they present a meta-analysis including 113 studies with a total sample of 11,525 patients, showing an improvement in individuals after the use of non-pharmacological measures to control associated respiratory pathology (weighted effect size, 0.30; 95% CI, 0.25-0.36; $p<0.001$).

The common presence of associated respiratory pathology in cancer patients is a factor that can cause changes in body composition, such as loss of muscle mass or an increase in visceral fat. This type of non-pharmacological intervention has been shown to be an important tool in improving symptoms and some parameters related to body composition. In the study by Madison et al, moderate exercise (bioelectrical impedance) over 12 weeks was associated with a reduction in body fat in colorectal cancer survivors. Another study showed that an 8-week exercise intervention increased skeletal muscle mass and decreased visceral fat in a group of head and neck cancer patients undergoing chemotherapy. Fernandez-Lao et al showed that a multimodal exercise programme in breast cancer patients led to a reduction in body fat and an increase in lean body mass in a group of breast cancer patients. However, despite the potential effects, there is little evidence of functional rehabilitation interventions that have assessed the effects on body composition in patients with associated respiratory pathology.

The scientific evidence and recent conclusions of expert meetings on cancer and the benefits of these psychosocial interventions, both in the prevention and treatment of the different clinical aspects of cancer survivors or patients undergoing cancer treatment, point to the existence of sufficient evidence to support their efficacy in addressing physical function, fatigue, quality of life, pain, anxiety and depressive symptoms, among others, related to cancer.

Non-pharmacological psychosocial interventions have therefore been shown to be even more effective than pharmacological interventions in addressing these associated symptoms, leading us to consider the bio-psychosocial approach and multidisciplinary intervention (oncology, nursing, physiotherapy, occupational therapy and medicine) as the global context of intervention. For this reason, aspects related to loss of function and pain associated with anxiety-avoidance disorders should also be assessed and addressed, which can be assessed using kinesiophobia scores.

The cognitive-behavioural model of fear of exercise suggests that patients with chronic pain or fatigue syndrome tend to avoid activity because they believe that activity is the cause of these symptoms, such as pain and fatigue.

Avoidance behaviour leads to even greater fear and symptoms, resulting in more pain or fatigue, which may extend to patients with associated respiratory pathology, so it is important to restore optimal activity levels and avoid loss of physical function and ability.

The choice of the most appropriate intervention setting is based on clinical complexity and the patient's ability to self-manage their situation. For this reason, and with the support of experts, our study proposes a supervised intervention in the home setting after hospital discharge, adapted to the specific situation of patients with associated respiratory pathology who have just been discharged from hospital. This is in line with recommendations to improve and increase access and adherence to a functional rehabilitation programme for these patients.

Supervised follow-up outside the healthcare setting, both in the community and at home, has been shown to be successful in previous trials. This is shown in a meta-analysis of 14 randomised controlled clinical trials in breast cancer survivors with supervised intervention by telephone or e-mail. In any case, despite the choice of the home setting for its feasibility in our study population, supervision and controlled follow-up of the intervention is still a guarantee of good results, as shown in another recent meta-analysis of 128 trials with a total of 13,050 cancer patients, where supervised programmes had greater effects on physical activity. Follow-up is important not only for the correct implementation of the intervention programme, but also for achieving high adherence to the programme, as shown in a review of 23 trials and 1372 patients.

The best results in patients surviving or undergoing cancer treatment have been obtained with multimodal exercise programmes that combine different types of exercise, mainly aerobic and strength training, in addition to other interventions such as reeducation in activities of daily living and health education, adapted to the patient's general condition and functional capacity.

For this reason, the investigators propose an interdisciplinary intervention, carried out by occupational therapists, nurses, physiotherapists and doctors specialised in this type of patient, aimed at improving conventional clinical practice and implementing a programme of functional re-education and environmental adaptation that implements conventional clinical practice, and which the investigators also consider to be an essential intervention in the follow-up of patients with associated respiratory pathology once they are discharged from hospital.

Conditions

Conditions: Cancer
Dyspnea
Functionality
Quality of Life
Functional Capacity

Keywords: Functional Status
Cancer
Dyspnea
Quality of Life
Occupational therapy

Study Design

Study Type: Interventional

Primary Purpose: Supportive Care

Study Phase: N/A

Interventional Study Model: Parallel Assignment

Number of Arms: 2

Masking: Single (Participant)
Subjects will also be blinded and will not know which group they have been allocated to and therefore which intervention they will receive. In order to minimise any contamination between groups, the assessment process will be carried out by external research staff who will perform the measurements and who have been previously trained and educated to avoid subjective bias in the process, as they will be unaware of the intervention group to which each elderly centre is assigned, so the clinical trial will have a masking of the blinded assessment by third parties.

Allocation: Randomized

Enrollment: 80 [Anticipated]

Arms and Interventions

Arms	Assigned Interventions
Experimental: FUNCTIONAL REEDUCATION AND ENVIRONMENTAL ADAPTATION PROGRAMME<br>prescription of reeducation in activities of daily living and the prescription of assistive devices and environmental adaptations, for one month from the first baseline assessment at the time of hospital discharge.	FUNCTIONAL REEDUCATION AND ENVIRONMENTAL ADAPTATION PROGRAMME<br>the prescription of reeducation in activities of daily living and the prescription of assistive devices and environmental adaptations, for one month from the first baseline assessment at the time of hospital discharge.
Active Comparator: Health education programme:<br>instructions and recommendations for maintaining an active and healthy life will be given as part of a health education programme. These will focus on the benefits of an active lifestyle and general guidelines to follow, as well as the importance of nutrition and hydration in a healthy lifestyle.<br>They will receive the dossier of instructions and recommendations of the health education programme, as in the experimental group.	Health education programme:<br>instructions and recommendations for maintaining an active and healthy life will be given as part of a health education programme. These will focus on the benefits of an active lifestyle and general guidelines to follow, as well as the importance of nutrition and hydration in a healthy lifestyle. They will receive the dossier of instructions and recommendations of the health education programme, as in the experimental group.

Outcome Measures

Primary Outcome Measure:

1. Activities of daily living
   Barthel Index

   [Time Frame: Baseline; 1 week (follow up); 2 weeks (final)]

Secondary Outcome Measure:

2. dyspnoea
   Medical Research Council Dyspnoea: Scores: (0, best - 4, worst)

   [Time Frame: Baseline; 1 week (follow up); 2 weeks (final)]

3. Health-related quality of life
   EuroQol 5-D questionnaire. Scores: ranging from 0 (worst imaginable state of health) to 100 (best imaginable state of health).

[Time Frame: Baseline; 1 week (follow up); 2 weeks (final)]

4. General Pain
   Visual Analogue Scale. Scores: ranging from 0 (lowest degree of pain) to 10 (highest degree of pain).

   [Time Frame: Baseline; 1 week (follow up); 2 weeks (final)]

5. Physical performance assessment
   Short Physical Performance Battery. The total SPPB score is the sum of the three sub-tests and ranges from 0 (worst) to 12. Changes of 1 point have clinical significance. A score below 10 indicates frailty and an elevated risk of disability as well as falls.

   [Time Frame: Baseline; 1 week (follow up); 2 weeks (final)]

6. pain/fatigue-related fear of movement
   Tampa Scale for Kinesiophobia. The results are obtained by means of a total raw score (which can range from 17 to 68) and two subscale scores.

   [Time Frame: Baseline; 1 week (follow up); 2 weeks (final)]

Eligibility

Minimum Age: 18 Years

Maximum Age:

Sex: All

Gender Based: No

Accepts Healthy Volunteers: No

Criteria: Inclusion Criteria:

- To have, among the reasons for admission, an anatomopathological diagnosis of newly diagnosed or relapsed oncological disease.
- To be admitted to the Oncology Department of the University Hospital of Salamanca.
- Moderate to severe dependency: Barthel Index score between 20 and 55 points.
- Sign an informed consent form authorising their voluntary participation in the study.

Exclusion Criteria:

- Cognitive impairment as assessed by the Mini-Mental State Examination (MMSE) of less than 24 points.
- Haemoglobin level of less than 10g/dl.

Contacts/Locations

Central Contact Person:

Central Contact Backup:

Study Officials:

Locations: **Spain**
Eduardo Jose Fernandez Rodriguez
Salamanca, Castilla Y Leon, Spain, 37002
Contact: Eduardo PhD Fernandez, PhD 923294500 edujfr@usal.es

IPDSharing

Plan to Share IPD:

References

Citations:

Links:

Available IPD/Information:
