# Supplementary material for: Impact of functional re-education and environmental adaptation in cancer patients with respiratory pathology: Study protocol
Source: PLoS One. 2025 Jan 9;20(1):e0313207. doi: 10.1371/journal.pone.0313207 (PMC11717268; doi:10.1371/journal.pone.0313207)
Supplement: S3 File — (PDF) [file pone.0313207.s003.pdf]

PROTOCOL AND PROJECT DESIGN: EFFECTS OF A FUNCTIONAL REHABILITATION AND ENVIRONMENTAL ADAPTATION PROGRAM ON DEPENDENCY LEVELS, DYSPNEA, FUNCTIONAL CAPACITY, QUALITY OF LIFE, AND CAREGIVER BURDEN IN CANCER PATIENTS WITH ASSOCIATED RESPIRATORY PATHOLOGY: STUDY PROTOCOL

In recent years, advances in cancer treatments, along with a strong focus on preventive strategies, have led to earlier diagnosis and improved understanding of oncological diseases, significantly increasing survival rates for cancer patients. As a result, the concept of "long-term survivor" has become increasingly important.

With this increase in survival and the subsequent rise in treatment lines, more side effects negatively impact aspects such as functionality and quality of life in patients. Some of these effects may include cancer-related fatigue, anxiety, or associated respiratory pathologies like dyspnea. Dyspnea, in particular, can present a significant health issue, with considerable limitations for individuals. In some patients with advanced cancer, dyspnea may be a clinical sign characteristic of the final stages of the disease. Approximately 41% of palliative care patients experience dyspnea, with 46% of them describing its intensity as moderate to severe. Moreover, the incidence of dyspnea increases considerably in patients with histopathological diagnoses of lung cancer, with studies reporting incidences higher than 73%.

Most patients perceive dyspnea as an uncontrollable limiting factor that leads to avoidance behaviors, increasing inactivity. This, in turn, negatively impacts their functionality, creating fear/avoidance patterns toward movement in cancer patients, similar to those seen in patients with chronic pain, chronic fatigue syndrome, or fibromyalgia. Patients with respiratory problems adapt to their symptoms by reducing activity levels, worsening their physical condition and exertion-related dyspnea, a phenomenon known as the "respiratory patient cycle."

To manage this, conventional clinical practice measures are typically implemented, primarily from a pharmacological perspective. However, we believe these measures are insufficient for achieving optimal symptom control. Despite current pharmacological treatments, the main symptom of respiratory pathologies—dyspnea—is not always adequately controlled, possibly due to its multifactorial nature. Most patients perceive dyspnea as an uncontrollable symptom, leading to avoidance behaviors that further reduce their activity levels, negatively impacting their functionality. Therefore, from an integrated care perspective, focusing not only on the symptom but on the patient as a whole, we have observed the necessity of employing additional interventions to help patients readjust to daily activities.

We have observed that cancer patients with respiratory pathologies adapt to their symptoms by reducing activity levels, leading to a worsening of physical condition and exertional dyspnea. This progressive inability results in loss of mobility, self-esteem, work capacity, and social relationships.

These data demonstrate that associated respiratory pathology is a common and often underestimated problem in oncology. It has been observed that individuals in this situation struggle to normalize their daily lives, either due to clinical deterioration or difficulty in applying what they learned during their hospital stay.

Regarding intervention measures, the NCCN panel highlights the importance of educational measures and energy conservation techniques as fundamental components of a comprehensive functional rehabilitation program. In addition to educational measures, the NCCN panel recommends the prescription of energy conservation techniques as useful for managing

associated respiratory pathology. This panel analyzes, on the one hand, a multicenter clinical trial involving 296 patients currently undergoing active treatment, which reported a decrease in symptom intensity after applying the program. On the other hand, they present a meta-analysis of 113 studies, with a total sample of 11,525 patients, showing improvements in patients following the use of non-pharmacological measures to manage associated respiratory pathology.

The frequent presence of associated respiratory pathology in cancer patients can lead to alterations in body composition, such as loss of muscle mass or increased visceral fat. Non-pharmacological interventions have proven to be an important tool for improving symptoms and some body composition parameters. In a study by Madison and colleagues, they observed a decrease in body fat after 12 weeks of moderate physical activity (bioelectrical impedance) in colorectal cancer survivors. Another study showed that an 8-week physical exercise intervention increased skeletal muscle mass and reduced visceral fat in a group of head and neck cancer patients undergoing chemotherapy. Fernández-Lao and colleagues demonstrated that a multimodal physical exercise program in breast cancer patients resulted in reduced body fat and increased lean body mass in a group of breast cancer patients. However, despite their potential benefits, there is little available evidence on functional rehabilitation interventions that have evaluated the effects on body composition in patients with associated respiratory pathology.

Scientific evidence and recent conclusions from expert meetings on cancer and the benefits of psychosocial interventions, both in prevention and treatment of various clinical aspects in cancer survivors or patients undergoing treatment, indicate sufficient evidence to support their efficacy in addressing physical function, fatigue, quality of life, pain, anxiety, and depressive symptoms, among others, associated with cancer.

Psychosocial non-pharmacological interventions have demonstrated effectiveness in managing these associated symptoms, even surpassing pharmacological interventions. This leads us to consider the biopsychosocial approach and multidisciplinary intervention (oncology, nursing, physiotherapy, occupational therapy, and medicine) as the global context for the intervention. Therefore, it is also essential to assess and intervene in aspects related to loss of function and pain associated with fear-avoidance disorders, which can be evaluated through degrees of kinesiophobia.

The cognitive-behavioral model of fear of movement suggests that patients with chronic pain or fatigue syndrome tend to avoid activity, believing it to be the cause of symptoms like pain and fatigue. Avoidance behavior leads to increased fear and more severe symptoms, resulting in more pain or fatigue. This concept can be extended to patients with associated respiratory pathology, making it important to restore optimal activity levels and prevent the loss of physical functions and capacities.

The selection of the most appropriate intervention setting depends on the clinical complexity and the patient's ability to self-manage their condition. Therefore, with expert support, our study proposes a supervised home-based intervention after hospital discharge, tailored to the specific circumstances of patients with associated respiratory pathology who have just been discharged. This aligns with recommendations for providing better access and adherence opportunities for these patients to a functional rehabilitation program.

Supervised follow-up outside the healthcare setting, both in the community and at home, has shown positive results in various previous studies. A meta-analysis of 14 randomized controlled trials in breast cancer survivors with telephone or email-supervised intervention showed good

outcomes. Regardless of the home-based setting, which is chosen for its feasibility in our study population, supervised and controlled follow-up of the intervention remains key to achieving positive results.

The best outcomes in cancer survivors or patients undergoing treatment have been achieved with multimodal physical exercise programs that combine various types of exercises, primarily aerobic activities and strength training, in addition to other interventions such as re-education in daily living activities and health education, which will be adapted to the patient's general condition and functional capacities. Therefore, we propose incorporating an interdisciplinary intervention, carried out by occupational therapists, nurses, physiotherapists, and medical specialists, aiming to improve conventional clinical practice and introduce a functional rehabilitation and environmental adaptation program that supplements conventional clinical practice. This is considered an essential intervention for the follow-up of patients with associated respiratory pathology after being discharged from their hospital stay.

5.2.2 PRACTICAL UTILITY AND POTENTIAL TRANSFERABILITY OF PROJECT RESULTS TO CLINICAL PRACTICE IN RELATION TO HEALTH

As mentioned earlier, associated respiratory pathology is one of the most common symptoms related to cancer and its treatment, considered one of the most enduring symptoms with the greatest impact on quality of life. Therefore, the results of this study could be quickly integrated into the care process for patients with this symptomatology, with the goal of normalizing their daily lives. This research project is part of the regional research and innovation strategy for smart specialization (RIS3) of Castilla y León, which sets its thematic priorities in health and social care, demographic change, and well-being, aiming to improve the quality of life for citizens by researching new strategies that facilitate independent living for chronically ill and/or dependent individuals at home. The proposed intervention includes professionals from various healthcare levels, forming a multidisciplinary team that would support the development and implementation of the intervention.

The intervention will be carried out without overloading the in-hospital clinical practice, achieving a dual benefit. On the one hand, the patient will continue their rehabilitation intervention with reduced exposure and risk of infections or overburdening their environment. On the other hand, the hospital will benefit from an effective intervention outside the care center, which is currently overburdened due to multifactorial causes.

AIM

HYPOTHESIS: Functional rehabilitation and environmental adaptation programs improve fatigue, pain, functional capacity, and quality of life in cancer patients with associated respiratory pathology.

OBJECTIVES: The primary objective of the study is to evaluate the effects of a functional rehabilitation and environmental adaptation program on the improvement of autonomy in cancer patients with associated respiratory pathology.

The secondary objectives are as follows:

1. Evaluate the effect of a functional rehabilitation and environmental adaptation program on activities of daily living, attention, executive functions, and quality of life parameters in cancer patients with associated respiratory pathology.

2. Assess the effect of a functional rehabilitation and environmental adaptation program on physical parameters in cancer patients with associated respiratory pathology, such as fatigue, pain, functional capacity, and body composition.
3. Describe if there is a correlation between levels of associated respiratory pathology and dependency levels in individuals.
4. Describe if there is a correlation between levels of dependency and kinesophobia levels in individuals.
5. Determine if there is a difference in terms of efficacy based on the oncological pathoanatomical diagnosis.

MATERIAL AND METHODS

DESIGN AND SETTING OF THE STUDY: Design: A randomized, stratified, prospective, longitudinal clinical trial with a fixed parallel assignment scheme of an experimental group and a control group.

SAMPLE/PARTICIPANTS: Participants: The reference population consists of cancer patients with associated respiratory pathology hospitalized at the time of inclusion. The study population will be selected through consecutive sampling with the following selection criteria.

STUDY SETTING: The study will be carried out in a mixed setting between specialized care at the University Hospital of Salamanca (CAUSA) (Medical Oncology Department) and the University of Salamanca (Occupational Therapy Teaching and Assistance Unit, UDATO).

PARTICIPANT SELECTION CRITERIA:

INCLUSION CRITERIA:

- Having a histopathological diagnosis of newly diagnosed or relapsed cancer as a reason for hospitalization.
- Being admitted to the Oncology Department of the University Hospital of Salamanca.
- Moderate to severe dependency level: Barthel index score between 20 and 55 points.
- Signing an informed consent authorizing voluntary participation in the study.

EXCLUSION CRITERIA:

- Cognitive impairment assessed with a Minimental State Examination (MMSE) score below 24 points.
- Hemoglobin levels lower than 10 g/dl.

WITHDRAWAL CRITERIA:

- Patient's death.
- Disease progression leading to a terminal state.
- Hospitalization at the time of home follow-up.
- Incomplete final assessment.

Randomization: Patients in the study population, after the initial interview with the research team and having met the selection criteria, will be randomly assigned to two groups: an intervention group (IG) and a control group (CG). The allocation sequence will be generated by

an independent researcher using the Epidat 4.2 software, with a 1:1 ratio. Participants will be randomized based on the order of their baseline assessment, and the randomization sequence will remain concealed until each patient is assigned to the appropriate group.

Blinding: The sequence, randomization, recruitment, and assignment work for each group (experimental or control) will be completed by research staff not involved in the evaluations or interventions, preventing potential biases in the study. Participants will also be blinded and unaware of the group to which they are assigned and the intervention they will receive. External research personnel trained in evaluation procedures will be incorporated to minimize any contamination between groups, ensuring third-party blind assessment. Additionally, the researchers responsible for the statistical analysis will be blinded, increasing the scientific rigor and quality of the study.

Sample size: The sample size estimation is based on the potential modification of one of the study's primary variables, the Barthel Index score. A pilot study with a similar population was used as a reference, where the Barthel Index score changed by 5.8 points. With these premises, accepting an alpha risk of 0.05 and a beta risk of 0.2 in a bilateral contrast, 40 subjects in each group are required to detect a difference of at least 5.8 units. A common standard deviation of 8.7 is assumed. A follow-up loss rate of 10% has been estimated. The sample size calculation was performed with the EPIDAT 4.2 software.

PROCEDURES AND DATA COLLECTION

Evaluations and study plan: Participants will be evaluated at three time points during the study: baseline evaluation (at the time of patient referral and before hospital discharge), follow-up evaluation (after 15 days), and final evaluation (one month after the initial evaluation, after the intervention).

The baseline evaluation will be conducted after recruitment and prior to randomization and group assignment. This initial evaluation includes recording independent variables and primary outcome variables, intervening variables, and several objective evaluation tests. After randomization, the corresponding intervention for each group will be carried out. Two subsequent evaluations, the follow-up evaluation and final evaluation, will be performed, during which participants will complete the same objective tests as in the initial evaluation. Results will be reported individually to those who requested to receive their information at the start of the study. All evaluations will be carried out by trained research personnel.

Description of the variables

Primary outcome variable: Activities of Daily Living (ADL): ADLs will be assessed using the Barthel Index (BI), whose version has been translated and validated into Spanish (36). The scale assigns a score based on the degree of dependency for performing a series of basic activities, with each item scored between 0 and 15 points. The global range can vary between 0 (completely dependent) and 100 points (completely independent).

Secondary Variables:

- **Assessment of associated respiratory pathology or dyspnea:** "Medical Research Council Dyspnea Scale (mMRC)" (17). It allows the patient to quantify their own dyspnea visually and simply, with 5 levels of severity.
- **Assessment of Health-Related Quality of Life (HRQoL):** Quality of life will be assessed using the EuroQol 5-D (EQ-5D) questionnaire, adapted and validated in the Spanish population (39).
- **Pain Assessment:** Pain will be evaluated using the Visual Analog Scale (VAS), widely used for pain assessment. It is represented as a 10 cm line, ranging from 0 = no pain to 10 = worst pain imaginable (39).
- **Physical performance evaluation:** The Short Physical Performance Battery (SPPB), validated in our setting for primary health care, predicts disabilities and adverse events, dependency, institutionalization, and mortality (40).
- **Evaluation of fear of movement related to pain/fatigue:** The Tampa Scale for Kinesiophobia (TSK), developed to assess fear of movement associated with pain, will be applied in its modified 11-item version (TSK-F), validated in cancer patients (41,42).
- **Caregiver burden assessment:** The Zarit Caregiver Burden Scale-Short Version, reduced by Gort A. and colleagues in 2005, consists of 7 Likert scale items scored from 0 to 5. Scores above 17 indicate caregiver strain (18).

Interventions

To describe the intervention, we must differentiate between the experimental group and the control group. Additionally, we should note that the research team will consist of occupational therapists, nurses, physiotherapists, and medical oncologists. Each professional will be responsible for their specific area of work:

CONTROL GROUP (CG):
Intervention for the control group: Delivery of an educational health program booklet prior to hospital discharge.

Educational Health Program: At the time of hospital discharge, after the baseline evaluation, instructions and recommendations for maintaining an active and healthy lifestyle will be provided, as will be done in the control group, as part of a health education program. These instructions will primarily address aspects related to the benefits of an active lifestyle and general guidelines to follow, as well as the importance of nutrition and hydration for a healthy life.
Participants will receive the booklet with instructions and recommendations from the health education program, as in the experimental group.

EXPERIMENTAL GROUP (EG):

Intervention for the experimental group: Delivery of an educational health program booklet prior to hospital discharge + prescription of assistive devices and home adaptations + functional rehabilitation.

Patients assigned to the intervention group will undergo a structured and supervised home program consisting of the health education program, combined with a **Functional Rehabilitation and Environmental Adaptation Program**, which will include the prescription of multimodal physical exercise, rehabilitation of activities of daily living (ADLs), and the prescription of

assistive devices and home adaptations. The intervention will last for one month from the time of the baseline evaluation at hospital discharge.

1. **Rehabilitation of Activities of Daily Living (ADLs):** Specific training will be conducted after the evaluation and prior to the patients' discharge from the university hospital. The goal is to identify factors that interfere with the performance of daily living activities. The intervention will consist of three parts: direct intervention on ADLs, teaching Energy Conservation Techniques (ECT), and providing advice on sleep hygiene measures, as outlined in the specific guidelines of the National Comprehensive Cancer Network (NCCN) (45).

   The intervention will consist of three parts: I. **Direct intervention on Activities of Daily Living (ADLs):** This will be carried out in the hospital setting and generalized to the patients' daily environments. The aim is to help patients become as autonomous as possible. The first session will take place the day before hospital discharge, with continued supervision throughout the month-long intervention.

   II. **Teaching Energy Conservation Techniques (ECT):** These techniques will be based on simplifying activities.

   ENERGY CONSERVATION TECHNIQUES FOR PATIENTS (ECT):

   1. Work areas where the activity is carried out should be organized.
   2. Work surfaces will be adapted, and objects should be within the patient's reach.
   3. When possible, activities should be performed in a seated position; standing will be encouraged as soon as feasible.
   4. Impulsive and vigorous movements should be transformed into studied, slow, and harmonious ones.
   5. Alternate heavy activities with light ones, incorporating rest periods between them.
   6. Balanced periods of activity will be combined with periods of rest.
   7. Avoid movements or activities that trigger exertion-induced dyspnea.

   Table 18. Energy Conservation Techniques for Cancer Patients (ECT)

   III. **Advice on Sleep Hygiene Measures:** As outlined in the specific guidelines of the National Comprehensive Cancer Network (NCCN).

   SLEEP HYGIENE MEASURES FOR CANCER PATIENTS:

   - Maintain as regular a sleep-wake rhythm as possible, particularly the wake-up time.
   - Avoid stimulant medications and other substances before sleep.
   - Maintain good nocturnal analgesic control, preferably using long-acting analgesics.
   - Avoid unnecessary time in bed during the day; for bedridden patients, provide physical and cognitive stimulation throughout the day.
   - Minimize nighttime interruptions from noise, medication administration, or other environmental factors.
   - Remove unpleasant stimuli, such as clocks, from the bedroom.

- Avoid staying awake in bed trying to sleep; engage in a relaxing activity (e.g., reading) outside of bed until sleepiness occurs.
- Avoid late afternoon naps.
- Use hypnotic medications after proper evaluation of sleep disorders and avoid overuse.

Table 19. Sleep Hygiene Measures for Cancer Patients

2. **Prescription of Assistive Devices and Environmental Adaptations:** Prior to patient discharge and after the baseline evaluation, the possibility of prescribing assistive devices to promote patient autonomy will be assessed, as well as identifying potential barriers in the home environment that could hinder autonomy after discharge. This will be done on the last day before hospital discharge and in the patient's home “in situ.”

WORK PLAN AND VISIT STRUCTURE

Once potential candidates to participate in the study are identified in the Oncology Department of the Complejo Asistencial Universitario de Salamanca (CAUSA), the researchers will conduct an interview to explain the purpose of the study and invite them to sign the informed consent form. Each participant will attend three evaluation visits: a baseline evaluation at the start of the study (before randomization) and two follow-up visits at 15 days and 1 month after the first visit. Each of the three scheduled visits will follow the same structure and last approximately one hour. Additionally, telephone follow-up will be conducted 3 and 6 months after randomization. During these visits, the study variables will be evaluated.

BASELINE VISIT:

This will take place just before the patients are discharged from the hospital. Initial data collection will include sociodemographic information, medical history, the presence of comorbidities, and the use of concomitant medications, ensuring that the selection criteria are met. All study variables and questionnaires will be evaluated and completed. At the end of the baseline visit, participants will be randomly assigned to one of the two study groups. For participants included in the experimental group, the structure and organization of the functional rehabilitation program will be explained during a specific visit.

FOLLOW-UP VISITS (15 DAYS) AND FINAL VISIT (1 MONTH):

These visits will be identical to the baseline evaluation except for the collection of sociodemographic variables, which will only be recorded during the baseline visit. The follow-up visits will take place at the Unidad Docente Asistencial de Terapia Ocupacional (UDATO) of the University of Salamanca.

DATA ANALYSIS

For the descriptive analysis of the data, normality will be checked using the Kolmogorov-Smirnov and Shapiro-Wilk tests (for $n < 30$). Variables with a normal distribution will be described by mean, standard deviation, and value range, while variables that do not follow a normal distribution will be described using the median and interquartile range. Qualitative variables will be presented as frequencies and percentages.

Regarding quantitative analysis, a correlation analysis (Pearson correlation coefficient) will be performed to demonstrate the validity of the evaluation procedure selected for our study. Cronbach's alpha coefficient will be used to demonstrate its reliability. For comparing two means, inferential statistical analysis will use the Student's t-test (parametric method for independent samples), the Mann-Whitney U test (non-parametric test for two independent samples), and the Wilcoxon test (non-parametric test for repeated measures). The comparison of three or more means will be analyzed using: ANOVA (for independent groups using Snedecor's F -ANOVA-) and Kruskal-Wallis (non-parametric method using the H test). For repeated measures, Snedecor's F test will be used in parametric testing and Friedman's test in non-parametric testing. Correlations will be performed using two methods: Pearson correlation (for normally distributed variables) or Spearman correlation (for non-normally distributed variables). A multivariate logistic regression analysis will be performed to determine variables associated with events of interest. In the logistic regression model, variables that were significant in the bivariate analysis or those relevant to the study will be included. For qualitative or categorical variables, contingency tables and the Chi-Square significance test will be used for two independent samples.

P-values less than 0.05 will be considered significant, that is, with a 95% confidence interval. IBM SPSS Statistics version 28.0.1 will be used for the statistical analysis.

The statistical analysis will be conducted by intention-to-treat. Population characteristics will be presented as means and standard deviations for continuous variables and as frequency distributions for qualitative variables. To evaluate comparability at baseline between the two study groups, the Chi-Square test will be used for qualitative variables, and the Student's t-test will be used for comparing means between the two groups. The effect of the intervention on the study variables will be assessed using a repeated measures ANOVA with two study factors (time * group). Subgroup analyses will be performed considering certain baseline evaluation variables and/or categories, such as age or initial score on the FACT scale. Hypothesis testing will have an alpha risk of 0.05 as the statistical significance threshold. The statistical software used will be SPSS, version 23.0.

ETHICAL ASPECTS

The study will be conducted after obtaining approval from the Ethics Committee for Clinical Research of the Salamanca Health Area, with informed consent from the study subjects and in accordance with the Declaration of Helsinki. Participants will be informed of the project's objectives and the risks and benefits of the procedures that will be performed (informed consent). Confidentiality of the subjects included will be guaranteed at all times in accordance with the provisions of Organic Law 3/2018 of December 5, on the Protection of Personal Data and Guarantee of Digital Rights, as well as Regulation (EU) 2016/679 of the European Parliament and the Council of April 27, 2016 (GDPR), and under the conditions set out in Law 14/2007 on Biomedical Research.

Significant modifications to the protocol (such as changes in evaluation tools, selection criteria modifications, or changes in the interventions) will be immediately communicated to the Ethics Committee.

Since this is a randomized clinical trial, it follows the CONSORT guidelines and has been registered. **TRIAL REGISTRATION**: ClinicalTrials.gov; ID: NCT06035263

RESEARCH TEAM:

Principal Investigator:

Dr. Eduardo José Fernández Rodríguez

Researchers:
Dr. Juan Jesús Cruz Hernández

Dr. María Isabel Rihuete Galve

ETHICAL STANDARDS:

The study will follow the principles of the Declaration of Helsinki and the Belmont Report to ensure compliance with applicable ethical standards for this research. All information regarding the results will be treated strictly confidentially. Both the center and the research team are responsible for handling the data and are committed to complying with the current data protection regulations, specifically Organic Law 3/2018, of December 5, on the Protection of Personal Data and Guarantee of Digital Rights, and Regulation (EU) 2016/679 of the European Parliament and the Council of April 27, 2016 (GDPR). Data collected for the study will be identified using a code, so no identifying information will be included, and only the research team will be able to link the data to you. The research team will analyze your data based on public interest and/or the legitimate purpose of achieving the study's objectives. Subsequently, your personal information will only be retained by the center for your health care and by the research team for other scientific research purposes if you have given your consent and if the law and applicable ethical requirements allow it.

If the results of the study are eligible for publication in scientific journals, no personal data of participants will be disclosed. You have the right to access, rectify, or cancel your data, and you can limit the processing of incorrect data, request a copy, or have the data you provided for the study transferred to a third party. To exercise your rights, or if the participant wishes to obtain more information about the processing of their personal data, they can contact the principal investigator of the study (details provided at the end of this document), the Data Protection Officer of the Regional Health Authority (dpd@saludcastillayleon.es), or our center (dpd.husa@saludcastillayleon.es). Furthermore, you have the right to contact the Data Protection Agency if you are not satisfied.

STATISTICAL ANALYSIS:

The statistical analysis will be conducted on an intention-to-treat basis. Population characteristics will be presented as means and standard deviations for continuous variables and as frequency distributions for qualitative variables. To assess comparability at baseline between the two study groups, the Chi-Square test will be used for qualitative variables, and the Student's t-test will be used for comparing means between the two groups. The effect of the intervention on the study variables will be evaluated using a repeated measures ANOVA with two study factors (time * group). Subgroup analyses will be conducted considering certain baseline evaluation variables and/or categories, such as age or initial score on the FACT scale. Hypothesis tests will have an alpha risk of 0.05 as the statistical significance threshold. The statistical software used will be SPSS, version 23.0.

LIMITATIONS:
The study follows all the recommendations of the CONSORT guidelines, but due to the nature of the intervention, the participants will not be blinded to the intervention.

BIBLIOGRAPHICAL REFERENCES.

1. Rodriguez EJF, Rihuete MI, Hernández JJC. Estudio aleatorizado sobre la influencia de un programa de rehabilitación integral funcional en el control de la astenia relacionada con la enfermedad oncológica. Med Paliativa. 2018;25(3):160–7.

2. Glaus A. [Fatigue and cancer--indivisible twins? A comparison between cancer patients, patients with diseases other than cancer and healthy people]. Pflege. 1994 Sep;7(3):183–97.

3. Mahoney fi, barthel dw. functional evaluation: The barthel index. Md State Med J. 1965 Feb;14:61–5.

4. Baztán JJ, del Molino J, Alarcón T, San Cristóbal E, Izquierdo G. Índice de Barthel: instrumento válido para la valoración functional de pacientes con enfermedad cerebrovascular. Rev Española Geriatria y Gerontol. 1993;28(1):32–40.

5. Cella D. The Functional Assessment of Cancer Therapy-Anemia (FACT-An) Scale: a new tool for the assessment of outcomes in cancer anemia and fatigue. Semin Hematol. 1997 Jul;34(3 Suppl 2):13–9.

6. Reitan RM. TMT, Trail Making Test A & B. South Tucson, AR Reitan Neuropsychol Lab. 1992;

7. Badia X, Schiaffino A, Alonso J, Herdman M. Using the EuroQol 5-D in the Catalan general population: feasibility and construct validity. Qual Life Res. 1998 May;7(4):311–22.

8. Ho K, Spence J, Murphy MF. Review of pain-measurement tools. Ann Emerg Med. 1996 Apr;27(4):427–32.

9. Collins SL, Moore RA, McQuay HJ. The visual analogue pain intensity scale: what is moderate pain in millimetres? Pain. 1997 Aug;72(1–2):95–7.

10. Guralnik JM, Simonsick EM, Ferrucci L, Glynn RJ, Berkman LF, Blazer DG, et al. A short physical performance battery assessing lower extremity function: association with self-reported disability and prediction of mortality and nursing home admission. J Gerontol. 1994 Mar;49(2):M85-94.

11. Ostir G V, Kuo Y-F, Berges IM, Markides KS, Ottenbacher KJ. Measures of lower body function and risk of mortality over 7 years of follow-up. Am J Epidemiol. 2007 Sep;166(5):599–605.

12. Gomez-Perez L, Lopez-Martinez AE, Ruiz-Parraga GT. Psychometric Properties of the Spanish Version of the Tampa Scale for Kinesiophobia (TSK). J Pain. 2011 Apr;12(4):425–35.

13. Ritchie JD, Miller CK, Smiciklas-Wright H. Tanita foot-to-foot bioelectrical impedance analysis system validated in older adults. J Am Diet Assoc. 2005 Oct;105(10):1617–9.

14. Thompson PD, Arena R, Riebe D, Pescatello LS. ACSM's new preparticipation health screening recommendations from ACSM's guidelines for exercise testing and prescription, ninth edition. Curr Sports Med Rep. 2013;12(4):215–7.

15. Campbell KL, Winters-Stone KM, Wiskemann J, May AM, Schwartz AL, Courneya KS, et al. Exercise Guidelines for Cancer Survivors: Consensus Statement from International Multidisciplinary Roundtable. Med Sci Sports Exerc. 2019 Nov;51(11):2375–90.

16. Mock V, Atkinson A, Barsevick AM, Berger AM, Cimprich B, Eisenberger MA, et al. Cancer-

related fatigue. Clinical Practice Guidelines in Oncology. J Natl Compr Canc Netw. 2007 Nov;5(10):1054–78.

17. Neefjes ECW, van den Hurk RM, Blauwhoff-Buskermolen S, van der Vorst MJDL, Becker-Commissaris A, de van der Schueren MAE, et al. Muscle mass as a target to reduce fatigue in patients with advanced cancer. J Cachexia Sarcopenia Muscle. 2017 Aug;8(4):623–9.

18. Keilani M, Hasenoehrl T, Baumann L, Ristl R, Schwarz M, Marhold M, et al. Effects of resistance exercise in prostate cancer patients: a meta-analysis. Support care cancer Off J Multinatl Assoc Support Care Cancer. 2017 Sep;25(9):2953–68.

20. Yen C-J, Hung C-H, Kao C-L, Tsai W-M, Chan S-H, Cheng H-C, et al. Multimodal exercise ameliorates exercise responses and body composition in head and neck cancer patients receiving chemotherapy. Support care cancer Off J Multinatl Assoc Support Care Cancer. 2019 Dec;27(12):4687–95.

21. Fernández-Lao C, Cantarero-Villanueva I, Ariza-Garcia A, Courtney C, Fernández-de-las-Peñas C, Arroyo-Morales M. Water versus land-based multimodal exercise program effects on body composition in breast cancer survivors: a controlled clinical trial. Support care cancer Off J Multinatl Assoc Support Care Cancer. 2013 Feb;21(2):521–30.
